# The impact of vaporized nanoemulsions on ultrasound-mediated ablation

**DOI:** 10.1186/2050-5736-1-2

**Published:** 2013-04-25

**Authors:** Peng Zhang, Jonathan A Kopechek, Tyrone M Porter

**Affiliations:** 1 Department of Mechanical Engineering, Boston University, 110 Cummington Street, Boston, MA, 02215, USA

**Keywords:** Phase-shift nanoemulsions, Vaporized perfluorocarbon droplets, High-intensity focused ultrasound, Thermal ablation, Polyacrylamide hydrogels

## Abstract

**Background:**

The clinical feasibility of using high-intensity focused ultrasound (HIFU) for ablation of solid tumors is limited by the high acoustic pressures and long treatment times required. The presence of microbubbles during sonication can increase the absorption of acoustic energy and accelerate heating. However, formation of microbubbles within the tumor tissue remains a challenge. Phase-shift nanoemulsions (PSNE) have been developed as a means for producing microbubbles within tumors. PSNE are emulsions of submicron-sized, lipid-coated, and liquid perfluorocarbon droplets that can be vaporized into microbubbles using short (<1 ms), high-amplitude (>5 MPa) acoustic pulses. In this study, the impact of vaporized phase-shift nanoemulsions on the time and acoustic power required for HIFU-mediated thermal lesion formation was investigated *in vitro*.

**Methods:**

PSNE containing dodecafluoropentane were produced with narrow size distributions and mean diameters below 200 nm using a combination of sonication and extrusion. PSNE was dispersed in albumin-containing polyacrylamide gel phantoms for experimental tests. Albumin denatures and becomes opaque at temperatures above 58°C, enabling visual detection of lesions formed from denatured albumin. PSNE were vaporized using a 30-cycle, 3.2-MHz, at an acoustic power of 6.4 W (free-field intensity of 4,586 W/cm^2^) pulse from a single-element, focused high-power transducer. The vaporization pulse was immediately followed by a 15-s continuous wave, 3.2-MHz signal to induce ultrasound-mediated heating. Control experiments were conducted using an identical procedure without the vaporization pulse. Lesion formation was detected by acquiring video frames during sonication and post-processing the images for analysis. Broadband emissions from inertial cavitation (IC) were passively detected with a focused, 2-MHz transducer. Temperature measurements were acquired using a needle thermocouple.

**Results:**

Bubbles formed at the HIFU focus via PSNE vaporization enhanced HIFU-mediated heating. Broadband emissions detected during HIFU exposure coincided in time with measured accelerated heating, which suggested that IC played an important role in bubble-enhanced heating. In the presence of bubbles, the acoustic power required for the formation of a 9-mm^3^ lesion was reduced by 72% and the exposure time required for the onset of albumin denaturation was significantly reduced (by 4 s), provided that the PSNE volume fraction in the polyacrylamide gel was at least 0.008%.

**Conclusions:**

The time or acoustic power required for lesion formation in gel phantoms was dramatically reduced by vaporizing PSNE into bubbles. These results suggest that PSNE may improve the efficiency of HIFU-mediated thermal ablation of solid tumors; thus, further investigation is warranted to determine whether bubble-enhanced HIFU may potentially become a viable option for cancer therapy.

## Background

High-intensity focused ultrasound (HIFU) is a medical procedure for the treatment of solid tumors [1-6]. In this procedure, ultrasound is focused into diseased tissue, and a fraction of the acoustic energy is converted into heat, primarily due to viscous absorption. Thermal ablation is possible by heating the tissue beyond the threshold temperature for protein denaturation. Using a focused transducer, the maximum point of heat deposition can be localized with millimeter precision. Thus, HIFU can be used to ablate solid tumors with minimal thermal damage to the surrounding and intervening tissue.

The focused ultrasound beam is normally generated with a single-element spherically focused transducer or by a transducer array. Because ultrasound can propagate through the tissue, the HIFU treatment does not require the insertion of probes and thus is noninvasive. In the past decade, HIFU has been used widely and has proven clinically to be successful in the treatment of a variety of cancers [7-9]. However, HIFU treatment of cancers that grow in organs behind by the rib cage or the skull (i.e., liver and brain cancers) is difficult because high attenuation of ultrasound in the bone increases the risk of thermal damage to the bone and the adjacent tissue. Additionally, acoustic intensity at the focus is reduced significantly, increasing the insonation time required for lesion formation. Therefore, a method that can reduce the acoustic power required for ablation and can increase the accuracy of the treatment while maintaining the therapeutic benefit will improve the clinical utility of HIFU for cancer therapy.

It has been well documented that the presence of microbubbles during sonication can increase the absorption of acoustic energy and can accelerate heating, which potentially could be used for increasing the efficiency of HIFU ablation [10-17]. While the results from documented studies are encouraging, microbubbles are not readily available in the tissue and thus must be created or introduced. Focused ultrasound can nucleate microbubbles in the tissue; however, it has been predicted that the applied rarefactional pressure must exceed 10 MPa in the absence of a nuclei [18,19]. At such a high pressure, shock waves can form in the tissue, and the absorption of shock waves may heat the tissue beyond the boiling point in milliseconds [20]. Because boiling bubbles can distort the lesion geometry significantly, the avoidance of boiling tissue is preferred during HIFU-mediated ablation [21,22]. The introduction of exogenous agents can serve as the nuclei for cavitation *in vivo*, thus reducing the pressure threshold. Studies have demonstrated that systemically administered ultrasound contrast agents (UCAs) can nucleate cavitation for bubble-enhanced heating [14,23-25]. However, the short lifespan of UCAs in circulation (<10 min) limits their use for bubble-enhanced thermal ablation [26,27]. Furthermore, UCAs located in the blood vessels along the acoustic propagation path may attenuate HIFU significantly, resulting in unwanted heating and thermal damage in the healthy intervening tissue. The feasibility of using laser-illuminated gold nanoparticles has also been demonstrated for nucleating cavitation [28]. However, this method was limited to superficial cancers due to lack of penetration of the laser in the tissue. Therefore, a reliable and consistent approach to nucleation locally within tumors is needed in order to take advantage of bubble-enhanced tumor ablation clinically.

In a previous study, we investigated the potential of using phase-shift nanoemulsions (PSNE) for nucleating microbubbles and reducing the pressure threshold for inertial cavitation (IC) [29]. PSNE consist of nanodroplets composed of liquid perfluorocarbon, such as dodecafluoropentane (DDFP), and coated with phospholipids or albumin. The boiling point of DDFP in bulk is 29°C at standard atmospheric pressure, which is lower than physiological temperature (37°C). However, when liquefied DDFP is dispensed in the form of a nanoemulsion, DDFP is stable in the liquid phase at temperatures approaching 70°C. This is most likely due to the surface tension, which creates a pressure difference across the droplet interface that is inversely proportional to the droplet radius (i.e., Laplace pressure). It has been predicted that as the internal pressure is increased, the boiling point is elevated [30,31]. In our previous study, we produced nanoemulsions with a mean diameter of 260 nm. Using the surface tension reported for naked perfluoropentane (PFP) droplets of 56 ± 1 mN/m [32], the Laplace pressure for our nanoemulsions was approximately 860 kPa. Nanoemulsions must be coated with surface-active molecules (i.e., surfactant) in order to inhibit fusion, and Rapoport et al. estimated that the coating may drop the surface tension for PFP to 30 mN/m [31]. Using the Antoine equation log_10_*P* = *A* − *B*/(*T* + *C*) [33] and the Antoine constants *A* = 6.87362, *B* = 1,075.780, and *C* = 233.205 which were previously reported for *n*-pentane [34], we calculated a boiling point of 90.8°C for our nanoemulsions.

While stable at physiological temperature, the PSNE can be vaporized with short (<1 ms), high-amplitude (>5 MPa) acoustic pulses, a process known as acoustic droplet vaporization (ADV) [35]. In a previous study, we used HIFU to vaporize PSNE dispersed throughout a polyacrylamide gel in a localized manner. The PSNE were vaporized only at the transducer focus when the rarefactional pressure exceeded a well-defined threshold (approximately 5 MPa); thus, HIFU provided a means to vaporize PSNE with exceptional spatial specificity and precision (i.e., on the order of millimeters). The PSNE reduced the pressure required for bubble formation in the gel phantoms and reduced the pressure for the onset of IC. Unlike UCA, scatter and attenuation from PSNE in liquid form are negligible; thus, unvaporized PSNE along the propagation path do not shield PSNE at the focus from the transmitted pulses. In addition, PSNE have no known toxicity, and DDFP has previously been tested clinically [36]. Thus, PSNE is a good candidate for localized bubble nucleation in the tissue.

The main purpose of this study was to examine the feasibility of using vaporized PSNE to accelerate HIFU thermal lesion formation. The study was performed using PSNE dispersed throughout optically transparent albumin-containing gel phantoms, where lesion formation could be visualized and analyzed with video techniques. Furthermore, an ultrasound protocol was designed specifically for vaporizing the PSNE and driving bubble-enhanced heating in a localized manner. For the ultrasound exposure, a short (<1 ms), high-amplitude acoustic pulse was transmitted first to trigger acoustic droplet vaporization followed by continuous wave (CW) sonication. The acoustic intensity of the continuous wave exposure was below the ADV threshold; thus, we anticipate that the vaporization of additional PSNE will be avoided, limiting the impact of bubbles on HIFU ablation to the focal volume. In addition, in this study, DDFP droplets were coated with a phospholipid shell instead of albumin, in order to achieve narrower size distributions through extrusion. A narrower size distribution enables more reliable control over PSNE vaporization, and the reduced size would potentially increase the amount of PSNE accumulation in tumors *in vivo* through enhanced permeability and retention effect [37]. We hypothesized that vaporized PSNE would significantly reduce the exposure time or acoustic power needed for lesion formation. Additionally, we explored the effect of inertial cavitation activity from vaporized PSNE on HIFU-mediated heating. Finally, the effect of PSNE concentration and acoustic intensity on final lesion shape and location was investigated.

## Methods

### Preparation of lipid-based phase-shift nanoemulsion

The phase-shift nanoemulsions consisted of DDFP (C5F12, CAS 678-26-2, Synquestlabs, Alachua, FL, USA) droplets, which were dispersed in saline and stabilized with a phospholipid monolayer shell. The shell components included 1,2-dipalmitoyl-*sn*-glycero-3-phosphocholine (DPPC, Avanti Polar Lipids, Alabaster, AL, USA) and the lipopolymer 1,2-distearoyl-*sn*-glycero-3-phosphoethanolamine-N-[methoxy(polyethylene glycol)-2000] (DSPE-PEG2000, Avanti Polar Lipid) in the molar ratio of 25:1. DPPC worked as an emulsifier to stabilize the emulsion from coalescence, and DSPE-PEG2000 served as a polymer brush, limiting the interaction between droplets that could lead to fusion [38].

The nanoemulsions were prepared by combining ultrasound emulsification and pressure extrusion methods in order to get the desired mean diameter and size distribution. In the first step, 5.0 mg DPPC and 0.8 mg DSPE-PEG2000 were mixed with chloroform in a round-bottom flask. After mixing, the chloroform was removed by evaporation under vacuum, leaving a dry thin lipid film. The film was re-hydrated with 9.95 ml of saline to form a lipid solution. In the second step, 0.05 ml DDFP was added to the 9.95 ml of phospholipid saline solution and then emulsified with an ultrasonic liquid processor (Model VC505, Sonic & Materials, Newtown, CT, USA) for 60 s. The solution was kept in an ice water bath during sonication to avoid DDFP evaporation. The sonication step produced perfluorocarbon droplets with a lipid shell which were stable at physiological temperature. In the last step, the emulsion was forced 16 times at 20°C through a polycarbonate membrane with 200-nm pores (Whatman, Kent, ME, USA) using an extruder (10-ml LIPEX extruder, Northern Lipids, BC, Canada), yielding a 10-ml narrowly distributed suspension of DDFP nanodroplets. The nanoemulsion was stored in a sealed vial and refrigerated until further use. The size distribution of the nanoemulsion was determined at 37°C with a particle size analyzer (Model 90 Plus, Brookhaven Instruments, Holtsville, NY, USA).

### Fabrication of tissue-mimicking phantom

All the tests of bubble-enhanced lesion formation were conducted *in vitro* using the albumin-containing acrylamide gel phantom originally developed by Lafon et al. [39]. Slight modifications were made in order to uniformly distribute PSNE into the phantom and get better lesion visualization of lesion formation via video recording. The volume of each phantom was 2.65 × 2.65 × 1.71 cm^3^. This size was sufficient for this study since the largest lesion that was produced was 1 cm in length. The phantom was prepared by first mixing 2.1 ml of acrylamide (A9926, 40% 19:1 acrylamide/bis-acrylamide solution, Sigma-Aldrich Corporation, St. Louis, MO, USA), 1.2 ml of 1 M Tris buffer pH 8 (trizma hydrochloride and trizma base, Sigma-Aldrich Corporation), 0.1 ml of 10% (*w*/*v*) ammonium persulfate solution (APS, Sigma-Aldrich Corporation), and 1.08 g of bovine serum albumin (BSA, A3059, Sigma-Aldrich Corporation) in water. The entire solution was degassed for 1 h at 40°C, and then, PSNE was added. The mixture was stirred gently to get a uniform distribution of nanodroplets, and TEMED (87689, Sigma-Aldrich Corporation) was added last to initiate polymerization (1 μl TEMED/ml phantom solution). The phantom was submerged in a 12°C water bath during polymerization to remove the heat generated by the exothermic reaction. The volume fraction of DDFP in phantoms was used to describe the concentration of PSNE added, assuming that no DDFP was lost during emulsification and polymerization of the gels. Six different PSNE volume fractions were used for these experiments: 0.000%, 0.004%, 0.008%, 0.012%, 0.016%, and 0.020% (*v*/*v*), where 0.000% was used as the control case.

APS and TEMED in the recipe served as the cross linker and free radical generator, respectively. BSA served as an indicator of HIFU thermal ablation, as it denatures and turns white with sufficient heating [23,39]. Since polyacrylamide gels are optically transparent, the denaturation of BSA was recorded real time using a hard disk drive camcorder with a 30-Hz frame rate (Everio, JVC, Yokohama, Japan). BSA also increases the attenuation coefficient of the gel. The speed of sound, density and attenuation of this type of phantom, as measured by Lafon et al. [39] at room temperature (22°C), were 1,044 ± 15 kg/m^3^, 1,544 ± 11 m/s, and 0.068 Np/cm at 3.2 MHz, respectively. All phantoms in this study were used on the same day of polymerization.

### Experimental setup

**Figure 1:**
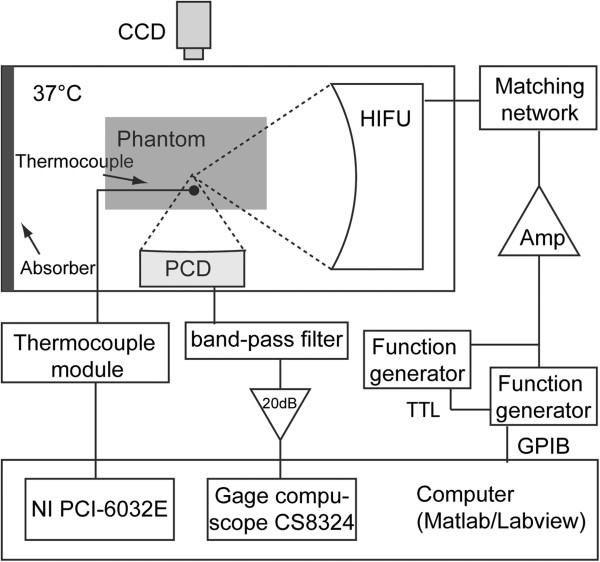
**Experimental setup.** A schematic of the setup for the polyacrylamide gel phantom experiments, indicating the relative positions of the HIFU transducer, the passive cavitation detector (PCD), the thermocouple, and the video camera. The *electrical connections* between equipment used in the experiments are also indicated in the figure.

The experimental setup is presented in Figure 1. All the tests were conducted in an optically transparent acrylic water tank with a volume of 20 × 30 × 20 cm^3^ (width × length × height). The tank was filled with degassed water (dissolved oxygen concentration of 30%) using a degassing filter (MiniModule, Membrana, Wuppertal, Germany), and the water temperature was maintained at 37°C using a water heater (Model VPT-107, Omega Engineering, Stamford, CT, USA). Due to the lack of water circulation during the experiments, it is likely that there was a depth-dependent temperature gradient in the water tank. Thus, the phantoms were placed at the same depth for each experiment in order to obtain consistent results. Two ultrasound transducers were used: one serving as the power transducer, and the other serving as the passive cavitation detector (PCD). These two transducers were positioned perpendicular so as to be confocal to each other to increase the signal-to-noise ratio of the PCD. The transducers were aligned by performing pulse-echo measurements with a 4-mm aluminum bead that was suspended in the water tank. The volume of the overlapping foci was very small (<0.1 mm^3^); thus, the PCD may not have been able to detect cavitation activity beyond the focal region. Nevertheless, it is expected that the majority of PSNE vaporization occurred within the focal volume; thus, the cavitation measurements were informative. The phantom was positioned at the focus of the power transducer in a custom-built acrylic holder, which had Tegaderm-covered windows on all sides to allow ultrasound transmission. A hard disk drive camcorder (Everio, JVC) with a 30-Hz frame rate was used to record the lesion formation from the outside of the water tank. All videos were recorded and processed after tests with an image processing code written in Matlab (Mathworks, Natick, MA, USA).

#### Power transducer calibration

The power transducer was a single-element spherically focused transducer with a 64-mm aperture and a 63-mm radius of curvature (Model H-102, Sonic Concepts, Woodinville, WA, USA). The power transducer was driven at its third harmonic (3.2 MHz), and the focal width and depth (pressure full width at half maximum (FWHM)) were 0.42 and 4.5 mm, respectively, at a temperature of 37°C. The excitation signal was provided by two function generators (33250A, Agilent, Santa Clara, CA, USA) in series with a 150-W RF amplifier (ENI A150, Rochester, NY, USA). As the desired waveforms in the tests were a short, high-amplitude pulse followed by CW exposure, two function generators were used. The first function generator delivered the ADV pulse and was triggered with the computer, while the second function generator was triggered by the first after the high-amplitude pulse was sent. A TTL delay circuit was used between the function generators to avoid overlap between the ADV pulse and CW exposure. The amplifier output impendence was matched to the transducer impedance via a matching network provided by the manufacturer. The acoustic power output of the transducer was calibrated with radiation force balance method as a function of the electrical input power [40], and the electrical power was measured with a power meter (E4419B, Agilent). The uncertainty of the measurements was 7%.

#### Passive cavitation detection

**Figure 2:**
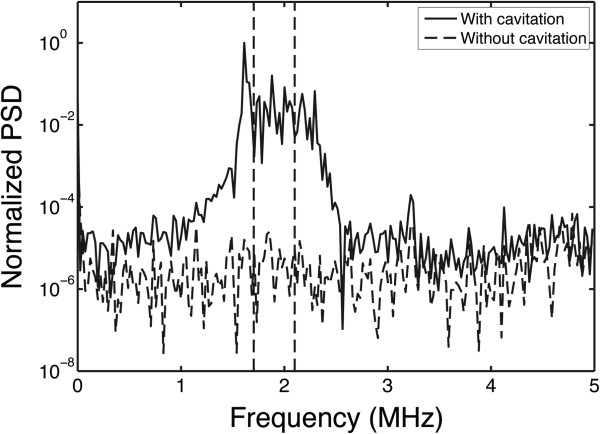
**A typical example of normalized PSD in gel phantom with vaporized and unvaporized PSNE.** The *solid line* represents the signal with vaporized PSNE, and the *dashed line* indicates the signal with unvaporized PSNE. The increase in broadband emissions around 2 MHz indicates the presence of inertial cavitation activity. Power density was integrated between 1.7 and 2.1 MHz and used as the IC dose of that segment. This frequency range was chosen due to the bandwidth of the 2-MHz PCD and to minimize the contribution from energy at the 1.6-MHz subharmonic peak.

A 2-MHz single-element spherically focused transducer with a 64-mm aperture and a 63-mm radius of curvature (Model H-106, Sonic Concepts) was used as a PCD to record cavitation emissions. The PCD had a focal width and depth (pressure FWHM) of 0.73 and 7 mm, respectively, at a frequency of 2 MHz. The signal from the passive transducer was filtered with a 1.6–2.2 MHz band-pass filter (Allen Avionics, Mineola, NY, USA) to suppress the fundamental frequency of the HIFU source. A low-noise preamplifier (Model DHPVA-100, Femto, Berlin, Germany) was used to amplify the filtered signal by 20 dB, and the amplified signal was digitized (14-bit dual-channel digitizer, Gage, Lockport, IL, USA) and stored on the computer. The sampling rate of the digitizer was set at 10 MS/s and recorded 2,024 data points every 5 ms. Figure 2 shows an example of the power spectrum of two segments, recorded with and without cavitating bubbles, respectively. The power density was integrated between 1.7 and 2.1 MHz and used as the IC dose of that segment. This frequency range was chosen due to the bandwidth of the 2-MHz PCD and to minimize the contribution from energy at the 1.6-MHz subharmonic peak. The noise floor was approximately four orders of magnitude below the cavitation signal.

### Experimental methods

#### Exposure conditions

The exposure conditions used in this study to evaluate bubble-enhanced heating and lesion formation are listed in Table 1. The transmitted acoustic power was measured using a radiation force balance, and the spatial average-temporal average acoustic intensity was approximated by dividing the measured acoustic power by the calculated FWHM cross-sectional area. All gels were sonicated with a 30-cycle tone burst followed by CW exposure for 15 s. PSNE-loaded gels were subjected to a tone burst with the acoustic power that was above (vaporization pulse (VP)) or below (non-vaporizing control pulse (NVP1)) the threshold for PSNE vaporization. Before commencing studies, we determined the pressure threshold for PSNE vaporization, which is dependent on the temperature, acoustic frequency, and pulse duration [41]. Using an acoustic method described previously to detect and quantify broadband emissions radiated by PSNE during vaporization [29], the free-field vaporization threshold for 3.3-MHz exposures at 37°C was determined to be 3.80 ± 0.27 W (*I* = 2,714 W/cm^2^) for a 30-cycle pulse. The vaporization threshold was identified by a nonlinear increase in the detected broadband emissions, as previously described [29].

**Table 1:** Ultrasound parameters: free-field acoustic powers and intensities to explore effect of PSNE vaporization on lesion formation

**Parameter name**	**Acoustic power (corresponding intensity)**	
	**Initial pulse (30 cycle)**	**Continuous signal (15 s)**
Vaporization pulse	6.4 W (*I* = 4586 W/cm^2^)	0.8 W (*I* = 550 W/cm^2^)
Heating (NVP1)	0.8 W (*I* = 550 W/cm^2^)	0.8 W (*I* = 550 W/cm^2^)
Heating (NVP2)	2.7 W (*I* = 1957 W/cm^2^)	2.7 W (*I* = 1957 W/cm^2^)

#### Temperature measurement with thermocouple

A needle thermocouple (0.2 mm, Model HYP-0, Omega Engineering, Stamford, CT, USA) was used in some experiments to measure temperature elevations during ultrasound exposures with and without PSNE vaporization. The thermocouple was inserted into the phantom at the HIFU focal plane, parallel to the HIFU axis and 0.63 mm off axis laterally, where the acoustic pressure was reduced by 67% compared to the pressure at the focus. The thermocouple was placed out of the axis plane of the two transducers to avoid interference with PCD measurements. The thermocouple signal was amplified (Model SCXI-1112, National Instrument, Austin, TX, USA), digitized (Model PCI-6035E, National Instrument), and stored in the computer. The system was calibrated as described previously [29], and the accuracy was determined to be ±0.3°C. The alignment of the thermocouple to the HIFU beam was conducted in two steps. First, the needle thermocouple was inserted into the polyacrylamide gel under the guidance of a B-mode ultrasound (Terason 2000, Terason Ultrasound, Burlington, MA, USA). Second, the power transducer provided a pulsed sinusoid signal with 50% duty cycle and 1 Hz pulse repetition frequency. The phantom was moved until a maximum rate in the temperature rise during the HIFU on time was measured (dT < 5°C) [16,42]. In the experiments, temperature elevation was measured using sonication parameters NVP1 and VP with a thermocouple inside a phantom mixed with 0.012% PSNE, and the corresponding PCD data were also recorded for comparison.

#### Monitoring lesion formation

All the tests were conducted with polyacrylamide gels separated into two groups. The first group was designed to test the feasibility of using vaporized PSNE to reduce the time or acoustic intensity required for lesion formation. The PSNE volume fraction was either 0.000% (sham), 0.008%, or 0.020%, and five tests were made for each volume fraction. In addition, four different acoustic intensities between 156 and 2,397 W/cm^2^ (with 7% uncertainty) were tested, with five tests at each intensity. All gel phantoms were sonicated with parameter VP (acoustic power of the tone burst exceeded vaporization threshold), NVP1 (acoustic power of the tone burst was below vaporization threshold), or NVP2 (acoustic power that is sufficient to cause lesion formation in the phantoms that did not contain PSNE). The second group was designed to investigate the effects of PSNE concentration in the lesion formation. Six different PSNE volume fractions were chosen (0.000%, 0.004%, 0.008%, 0.012%, 0.016%, 0.020%), and for each volume fraction, four or five sonications were made with parameters VP and NVP1. For all tests, the PCD and video data were recorded, stored, and processed later.

### Image processing and statistics of lesion geometry

**Figure 3:**
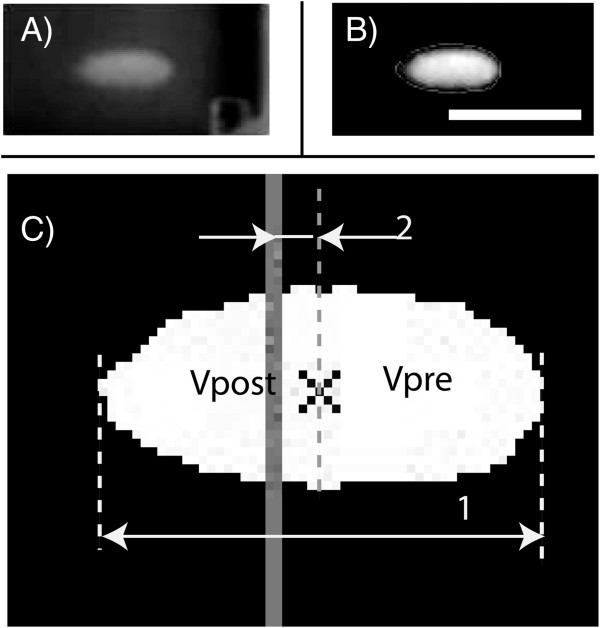
**An example of the image processing procedure (A, B, C).***1* represents the lesion length and *2* represents the shift of lesion center with respect to focal plane (*solid gray line*). The lesion was divided into two parts by its middle point, and the volume of each part was calculated (*V*_*Pre*_ and *V*_*Post*_). The ultrasound source was located to the *right* of the image. The *scale bar* represents 5 mm.

All videos were analyzed using an image processing code written in Matlab. Using the code, the HIFU on/off time, location of the focal plane and lesion, and the geometrical lesion dimensions were obtained. The focal plane was determined each day by identifying the center of a symmetric lesion in a phantom without PSNE, and this location was used as the focal plane for all other experiments conducted that day. The key algorithm in this code was lesion detection, which was designed based on background subtraction with suitable noise filtration methods. Figure 3 shows an example of how the code worked for each frame. First, the location of lesion in the video was detected and cropped (Figure 3A), and then the background was removed by subtracting a frame which was acquired before the sonication began. Next, the image was smoothed with a Gaussian filter and rescaled linearly for better image quality (Figure 3B). In addition, the position of the focal plane was calculated. The background noise threshold was determined by measuring the peak gray-scale pixel intensity in a video of a phantom without ultrasound exposure. All other acquired videos were thresholded using this value in order to identify the lesion boundary. Because only 2D information about lesions was available from the images, lesions obtained in this study were assumed to be axially symmetrical. This approximation was verified during the experiments by visually examining the lesions after sonication. Finally, the lesion dimensions, including length and center location relative to the focal plane, and the distortion (i.e., degree of prefocal lesion formation) in lesion shape along the HIFU axis were determined from the processed images (Figure 3C).

The lesion volume was estimated using a method previously described [23,43]. Assuming that the lesions were symmetrical around the long axis, the volume (*V*) could be approximated according to the following equation:

(1) $$V=\sum_{i=1}^{N}\pi \times {\left(r_{i}\right)}^{2}\times C_{p},$$

where *N* is the total number of pixels along the axis, *r* is the distance in pixels from the lesion border of that slice to its central axis, and *C*_*p*_ is the length of each pixel in millimeters. It has been reported that the presence of cavitation or boiling will cause lesion distortion along the HIFU axis [21,22]. By assuming that the lesion was divided into two parts with its middle point along the axis, the volumes of the proximal and distal parts of the lesion relative to the transducer were calculated as *V*_pre_ and *V*_post_, respectively. A distortion coefficient was then defined as *V*_pre_/*V*_post_ to represent the degree of distortion. If no distortion occurred, the lesion had a cigar shape and the coefficient was approximately equal to one. If distortion did occur, the lesion had a teardrop shape, resulting in a distortion coefficient greater than one.

## Results

### Size distribution of nanoemulsions

**Figure 4:**
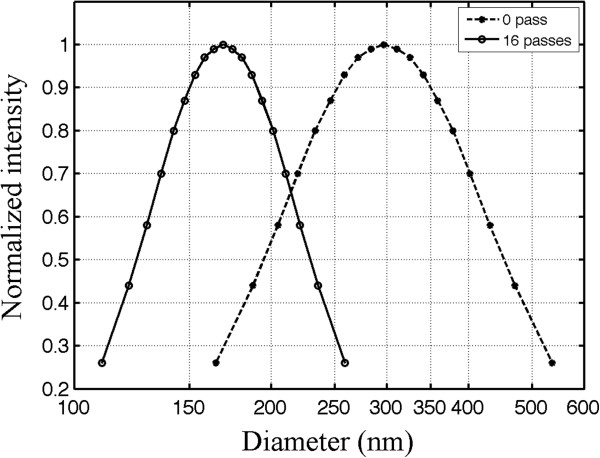
**The size distribution of nanoemulsion particles before and after extrusion.** The *dashed line* represents the distribution before extrusion, and the *solid line* indicates the distributions after extruding 16 times through polycarbonate membrane filters with a 200-nm pore size. Extrusion produced PSNE with a smaller mean diameter and a narrower size distribution. The measurement uncertainty was 1.1% (calculated from the variation in mean size after repeating the measurement five times with the same sample).

An example of the size distribution measured for the nanoemulsion is shown in Figure 4 (16 passes, solid line). The size distribution of the nanoemulsion before the extrusion is also shown in Figure 4 (0 passes, dashed line). Comparing the results before and after extrusion, we find that forcing the nanoemulsion 16 times through a membrane with a 200-nm pore size helped to reduce the polydispersity in the size distribution. Furthermore, the results suggest that the mean size of the nanoemulsion post-extrusion depends upon the membrane pore size. Therefore, the extrusion technique is extremely useful in the preparation of nanoemulsions where a predetermined particle size is desired. The mean diameter for all nanoemulsions extruded through the 200-nm membranes in this study was 173 nm with a standard deviation of 5 nm. A previous study found that microbubbles formed from vaporized droplets expanded in size six to ten times the original droplet diameter [44]. Thus, it is anticipated that the droplets used in this study would form micron-sized bubbles after vaporization.

### Temperature and passive cavitation detection

**Figure 5:**
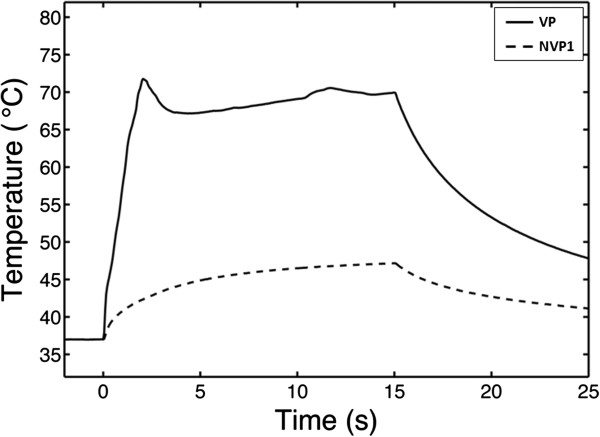
**A typical example of temperature in PSNE-containing gel phantom with and without a vaporization pulse.** A polyacrylamide gel phantom containing PSNE (volume fraction of 0.012%) was sonicated with VP (*solid line*) or NVP1 (*dashed line*) exposure conditions (see Table 1). The thermocouple was located 0.63 mm off axis laterally, where the acoustic pressure was 67% of the pressure at the focus. Ultrasound was transmitted continuously at an intensity of 550 W/cm^2^ between *t* = 0 s and *t* = 15 s. Vaporization of PSNE significantly accelerated heating in the gel phantom as compared to phantoms with unvaporized PSNE.

**Figure 6:**
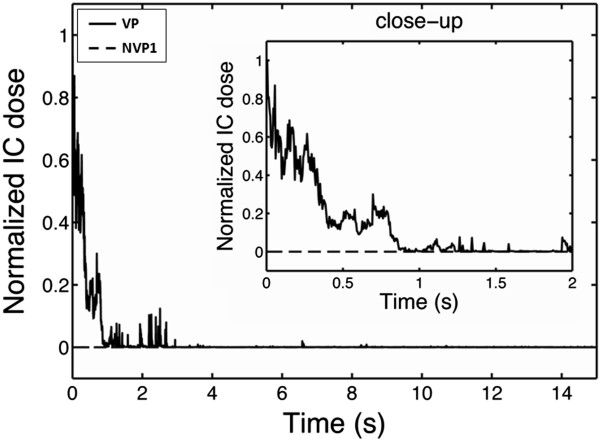
**Inertial cavitation dose as a function of time with and without a vaporization pulse.** The inertial cavitation dose, averaged between 1.7 and 2.1 MHz and normalized by the initial cavitation dose measured at the onset of sonication with PSNE, is plotted for the tests shown in Figure 5. Note that the time is plotted on a logarithmic scale. The peak temperature rise in Figure 5 corresponded to the time when the inertial cavitation dose was at its peak (within 1 s of vaporization).

Figure 5 shows the temperature elevation with and without PSNE vaporization in a phantom loaded with PSNE at a volume fraction of 0.012%. For these tests, gel phantoms were subjected to a CW exposure at an acoustic intensity of 550 W/cm^2^ for 15 s. In all tests, CW exposures were preceded by a 30-cycle tone burst at an acoustic power above (VP) or below (NVP1) the PSNE vaporization threshold. For gels in which PSNE was vaporized, the temperature rose significantly in the first 2 s. The temperature then dropped a few degrees and remained level through the remaining sonication time. An example of the inertial cavitation dose (normalized by the initial cavitation dose measured at the onset of sonication with PSNE) is plotted as a function of the sonication time in Figure 6. No evidence of inertial cavitation was detected for gels sonicated without PSNE vaporization (NVP1), while strong IC emission was detected immediately after PSNE vaporization (VP). However, the magnitude of broadband emissions dropped significantly in the first 2 s and was negligible for *t* > 3 s. This trend was observed in all tests where PSNE were vaporized. By comparing Figures 5 and 6, we can see a relationship between the temperature elevation and the IC dose. The observed acceleration in heating coincided in time with the presence of broadband emissions from inertial cavitation. Once the IC dose reached zero, the temperature measured with the needle thermocouple during HIFU exposure increased by only a few degrees. This suggests that inertial cavitation contributed significantly to the accelerated heating measured after vaporized PNSE, which is consistent with findings from previous studies [10,11,45].

### Effect of vaporized PSNE on HIFU-mediated lesion formation

**Figure 7:**
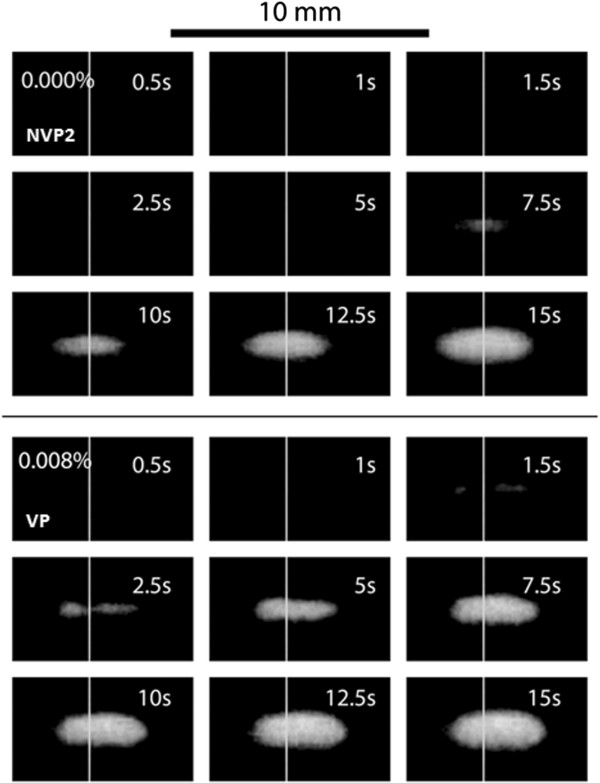
**Image sequences of lesion formation in phantoms with PSNE concentrations of 0.000% and 0.008%.** The control phantom (PSNE concentration of 0.000%) was sonicated with a 30-cycle tone burst followed by CW for 15 s, and the acoustic intensity was constant at 1,957 W/cm^2^. The phantom with 0.008% PSNE was sonicated with VP exposure condition (see Table 1). Lesions formed more rapidly in gel phantoms containing PSNE. The *white vertical line* on each image represents the focal center.

**Figure 8:**
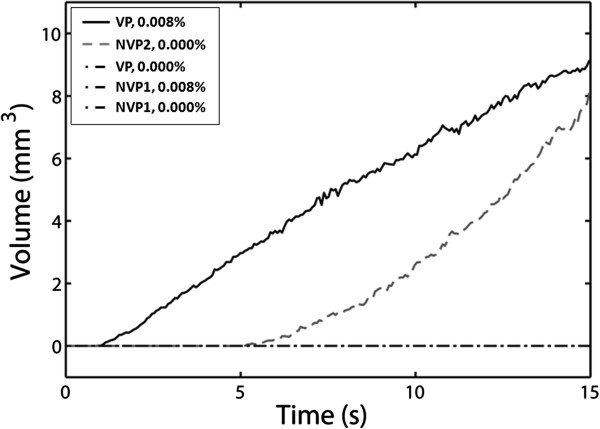
**Rate of growth in lesion volume as a function of PSNE volume fraction and ultrasound exposure conditions.** The ultrasound exposure conditions are listed in Table 1. Lesion formation in gel phantoms containing vaporized PSNE was enhanced compared to that in controls.

Images of lesion formation are shown over a 15-s exposure at PSNE volume fractions of 0.000% (control) and 0.008% in Figure 7. The onset of lesion formation was 2.5 s with PSNE compared to 7.5 s without PSNE. Also, a plot of the change in lesion volume over time for different combinations of exposure conditions (see Table 1) is shown in Figure 8. It is shown that the onset of lesion formation started earlier (i.e., 1.5 s) in a gel at a lower intensity (i.e., 550 W/cm^2^) when PSNE were vaporized before CW exposure. In comparison, evidence of the onset of lesion formation in gels without PSNE and subjected to more intense ultrasound (i.e., 1,957 W/cm^2^) was not seen until 7.5 s after the start of sonication. Another noteworthy result from this part of the study was the fact that lesions formed at an acoustic intensity of 550 W/cm^2^ after vaporization of PSNE at a volume fraction of 0.008% still had a symmetric shape (Figure 7).

### Effect of PSNE concentration on lesion formation

**Figure 9:**
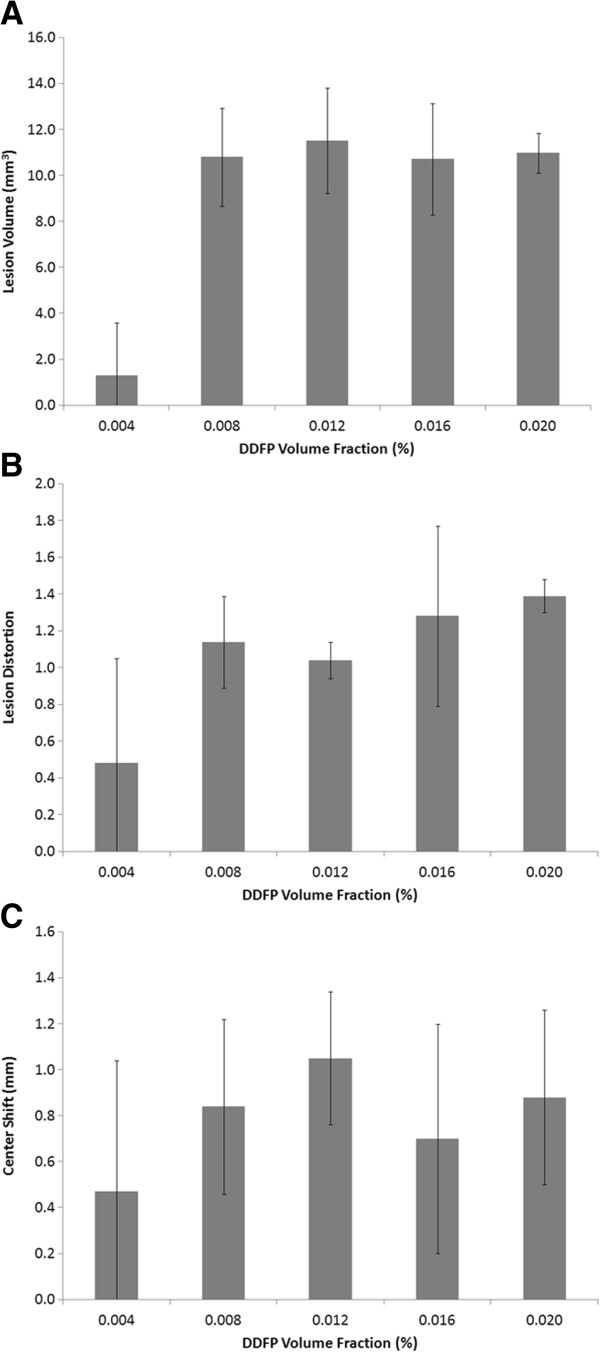
**Effect of PSNE volume fraction on lesion formation.** (**A**) Volume, (**B**) distortion, and (**C**) center shift of lesions as a function of PSNE volume fraction at an acoustic intensity of 550 W/cm^2^ with VP exposure condition (see Table 1). No statistically significant differences were observed between lesions formed with PSNE volume fractions above 0.004%. *Error bars* represent the standard deviation from five measurements.

The effect of PSNE concentration (i.e., DDFP volume fraction) on lesion volume, distortion, and center shift is plotted in Figure 9 (*N* = 5). All the results shown in this section were from tests performed with VP exposure conditions. Although tests with NVP1 exposure conditions were also conducted, no lesions were formed, and thus, no measurements could be made. The results show that either no lesion or small lesions (volume < 4 mm^3^) were formed with 0.004% PSNE. This suggests that a minimum density of cavitation nuclei or a longer CW exposure time is needed to take advantage of bubble-enhanced heating at this PSNE concentration and acoustic intensity. At an applied acoustic intensity of 550 W/cm^2^, there was no significant difference in the size, shape, and variance of lesions formed in gels containing 0.008% to 0.020% PSNE.

### Effect of acoustic intensity on lesion formation with vaporized PSNE

**Figure 10:**
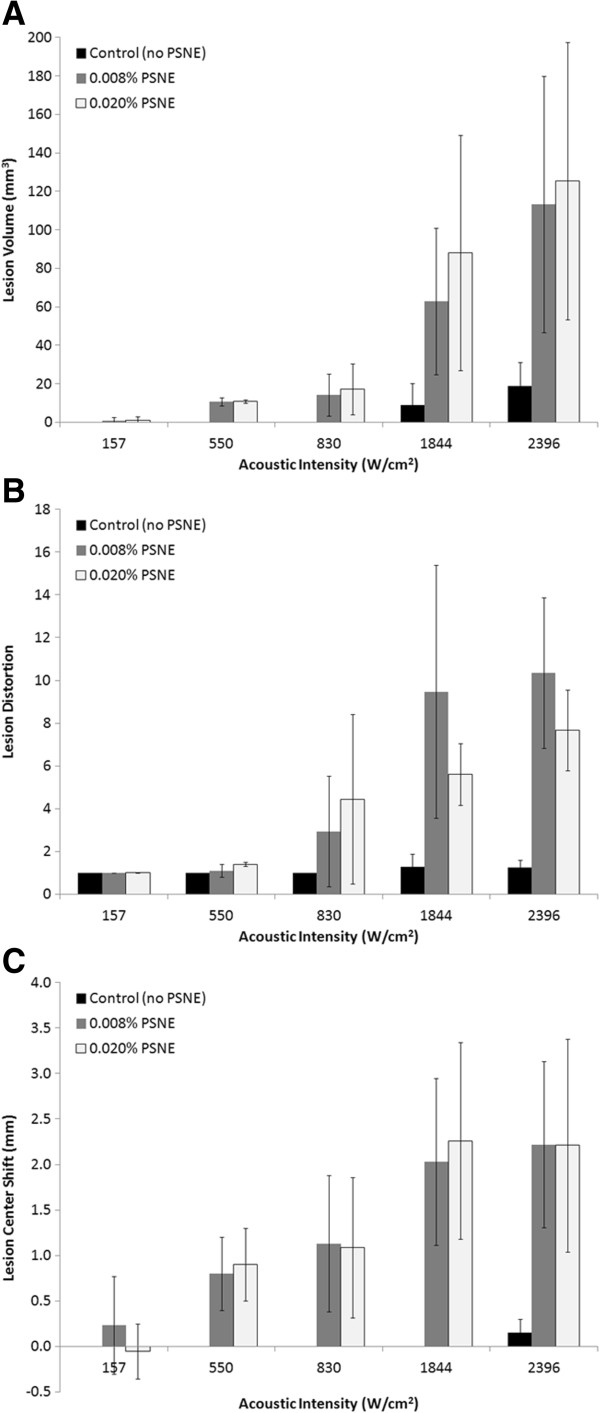
**Effect of acoustic intensity on lesion formation.** (**A**) Volume, (**B**) distortion, and (**C**) center shift of lesions as a function of continuous wave acoustic intensity. All samples were initially insonified with a 30-cycle, 6.4 W vaporization pulse. The acoustic intensity had a significant effect on the lesion volume, distortion, and center shift in gel phantoms containing PSNE. *Error bars* represent the standard deviation from five measurements.

**Figure 11:**
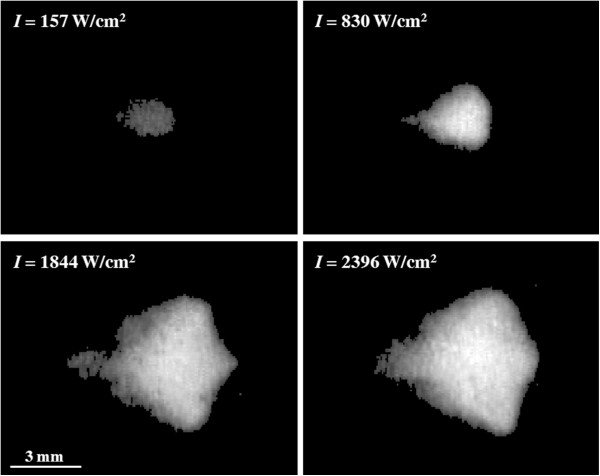
**Comparison of lesion geometries at different acoustic intensities.** It can be seen that the lesion becomes asymmetric at higher intensities. The PSNE volume fraction was 0.020% for all lesions.

**Figure 12:**
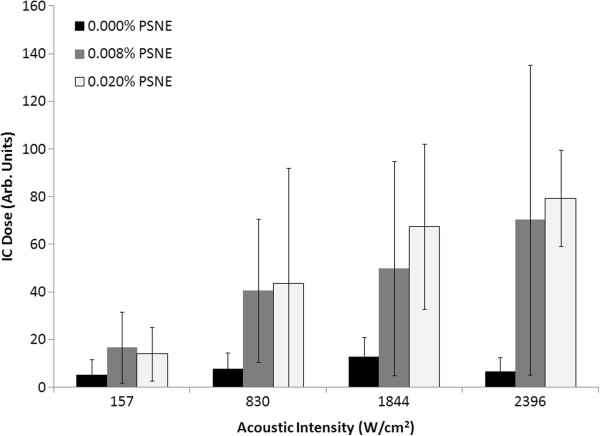
**Inertial cavitation dose at different acoustic intensities and PSNE concentrations.** The inertial cavitation dose corresponds to the tests in Figure 10. The inertial cavitation dose increased with acoustic intensity and was greater in gel phantoms that contained vaporized PSNE. *Error bars* represent the standard deviation from five measurements.

The lesion volume, distortion, and center shift are plotted as a function of continuous wave acoustic intensity at PSNE concentrations of 0.000% (control), 0.008%, and 0.020% in Figure 10. The lesion volume increased with acoustic intensity and was significantly enhanced by PSNE. We determined that the minimum acoustic intensity required to denature albumin in 15 s without vaporized PSNE was 1,479 W/cm^2^ (the volume of denatured albumin in this case was not measurable) compared to a minimum acoustic intensity of 157 W/cm^2^ with PSNE (uncertainties of 7%). Thus, it was possible to denature albumin in 15 s using 89% less acoustic power by first vaporizing PSNE. In general, the distortion and center shift were larger at higher intensities, although no difference was observed at acoustic intensities between 1,844 and 2,396 W/cm^2^. In addition, no statistically significant differences in the lesion sizes and shapes were observed for PSNE concentrations of 0.008% and 0.020%. Representative images of lesions formed at different continuous wave acoustic intensities are shown in Figure 11 for a PSNE concentration of 0.020%. The lesion geometry was very symmetric at an acoustic intensity of 157 W/cm^2^, but the lesions become asymmetric at acoustic intensities of 830 W/cm^2^ and greater. The inertial cavitation dose is plotted in Figure 12 as a function of continuous wave acoustic intensity for the conditions tested in Figure 10. The detected inertial cavitation dose increased with acoustic intensity and was greater for phantoms containing vaporized PSNE.

## Discussion

While this study is a continuation of our research on nucleating bubbles with PSNE for the enhancement of ultrasound-mediated thermal ablation, we have made significant modifications to the protocol for making the nanoemulsions in order to produce them at a more optimal size for future *in vivo* applications. The addition of extrusion to the protocol narrowed the size distribution and reduced the mean diameter of the nanoemulsions (Figure 4). For this study, nanoemulsions were produced with a mean diameter below 200 nm. This is advantageous for *in vivo* applications since sizes below 200 nm are optimal for passive accumulation in tumors through the enhanced permeability and retention effect [37]. Additionally, we coated the nanoemulsions with a mixture of phospholipids instead of albumin, which was used as the emulsifier in our previous study. Phospholipids can be conjugated with poly(ethylene glycol) (PEG), a polymer which has been shown to limit liposome aggregation and maintain a well-defined size distribution [46,47]. More importantly, it has been shown that PEG increases the circulation time of systemically administered liposomes *in vivo*[48-50]. Thus, we speculate that coating our nanoemulsions with PEGylated lipids will increase the circulation time *in vivo*, and this is the subject of an ongoing study. PSNE have no known toxicity, and the components of PSNE (DDFP and PEGylated lipids) have previously been tested clinically [36,51].

The primary objective of this study was to investigate the effect of vaporized PSNE on the time or acoustic power required for lesion formation. Studies were conducted with albumin-containing polyacrylamide gel phantoms because it allowed for control of the concentration of PSNE added as well as real-time visual observation of lesion formation. Variations in the lesion dimensions between experiments were observed with phantoms containing PSNE. Although care was taken during phantom preparation to ensure that the PSNE were well mixed within the acrylamide solutions, it was challenging to produce a perfectly uniform distribution of PSNE within the gel phantoms. Even tiny differences in the PSNE distribution within the phantoms can have a significant effect on the cavitation activity and thus heating rates, which can cause variation in lesion formation. For this reason, five experiments were performed for each condition tested. First, we confirmed that vaporized PSNE could nucleate inertial cavitation and accelerate HIFU-mediated heating within the phantom above the albumin denaturation temperature threshold. When PSNE were vaporized before CW exposure, the peak temperature measured outside the focal volume exceeded 70°C within 5 s (Figure 5). Provided the PSNE volume fraction was at least 0.008%, albumin denaturation was observed within 5 s in hydrogels treated after PSNE vaporization (Figure 9). More notably, it was possible to form a lesion of measurable volume (9 mm^3^) after PSNE vaporization (0.008% volume fraction) using 72% less power than the minimum required to denature albumin without vaporized PSNE (550 vs. 1,957 W/cm^2^, respectively). Furthermore, we found that the minimum acoustic intensity to denature albumin within 15 s is 89% less after PSNE vaporization (157 vs. 1,479 W/cm^2^). The reduction in acoustic power for lesion formation may have a significant impact on clinical applications of bubble-enhanced HIFU for cancer therapy, in particular for the treatment of brain and liver tumors. This reduction in power percentage (72%) to form measurable lesions exceeds the reduction in power percentage (30%) reported from a study of the effect of UCA on ultrasound-mediated thermal ablation in polyacrylamide gels [23]. The difference in the reduction in power percentage is most likely due to attenuation of the transmitted acoustic waves in the gel by UCA prefocally. In our study, bubbles are not present along the beam path and the attenuation of PSNE is negligible compared to the polyacrylamide gel. Therefore, it is possible to localize the effect of bubbles on ultrasound-mediated heating and thermal ablation by vaporizing PSNE *only* at the transducer focus.

Lesions formed after PSNE vaporization had a predictable symmetric cigar shape at acoustic intensities between 157 and 550 W/cm^2^. However, at intensities greater than 830 W/cm^2^, the lesions formed a teardrop shape similar to other studies of lesions formed in gels and tissue in the presence of bubbles [12,21,52]. Based upon these observations, there may potentially be an optimal range of acoustic intensities that allow for taking advantage of bubble-enhanced heating while avoiding distortion in lesion shape. The symmetry and geometry of lesions formed at acoustic intensities of 157 W/cm^2^ (Figure 11) and 550 W/cm^2^ (Figure 7) were comparable to the lesions formed without PSNE vaporization. The symmetric cigar shape is advantageous for planning bubble-enhanced HIFU tumor ablation as it makes it possible to predict the lesion shape. However, it is important to note that lesions formed in the presence of vaporized PSNE still migrated towards the transducer, which must be accounted for in treatment planning. In addition to maintaining symmetry in lesion shape at low acoustic intensities (<550 W/cm^2^), the lesion volume was comparable in gels containing 0.008% and 0.020% PSNE. This was unexpected as several studies have reported an increase in the volume ablated by ultrasound in the presence of bubbles [24,25,53]. For example, Kaneko et al. reported that the volume of lesions formed in tumors after systemic administration of Levovist was 371 ± 104 mm^3^ compared with 166 ± 71 mm^3^ for saline [54]. As an alternative to ultrasound contrast agents, Sokka et al. used a 0.5-s, 300-W tone burst to nucleate bubbles at the focus in a rabbit thigh [12]. In our study, the lesion volume was primarily determined by the volume in which PSNE were vaporized. Thus, increasing the PSNE concentration above 0.008% had no detectable effect on lesion volume created at the aforementioned acoustic intensities.

Although the vaporized PSNE were localized to the HIFU focal plane, the lesions formed due to bubble-enhanced heating did tend to grow towards the transducer. Documented studies show that boiling may alter the lesion location due to the backscatter of incident waves by millimeter-sized bubbles [22,55,56]. Nonlinear wave propagation may also move the peak HIFU intensity towards the transducer, resulting in growth of the lesion towards the transducer [57]. However, we did not observe any center shift for lesions generated without PSNE (Figure 7), which suggests that a shift in the lesion center most likely was not due solely to nonlinear wave propagation. While a prefocal shift in the location of maximum acoustic intensity due to nonlinear wave propagation may not be responsible for the migration of the lesion; it may shift the location of PSNE vaporization. Consequently, the impact of microbubbles on HIFU-mediated heating will be shifted towards the transducer, leading to the formation of lesions in the prefocal region. Another factor is that heating can induce changes in the acoustic attenuation of the gel phantom which could shift the focal region. Further studies on this topic are warranted as a sound fundamental understanding of the impact of cavitating bubbles on lesion location, size, and shape which is essential to the clinical translation of the technique for cancer therapy.

## Conclusions

In conclusion, the feasibility of using PSNE to accelerate HIFU thermal lesion formation in albumin-containing gel phantoms was demonstrated. Lipid-coated phase-shift nanoemulsions were produced with a narrow size distribution (between 100 and 300 nm), which is important as the pressure threshold for vaporizing PSNE depends upon the droplet size. When driven to cavitate inertially, the bubbles formed by vaporizing PSNE reduced the acoustic intensity required for lesion formation in gel phantoms by as much as 89%. In addition, at an acoustic intensity of 550 W/cm^2^, the onset of lesion formation was reduced from 5 to 1 s of insonation. Furthermore, symmetrical lesions can be formed in the presence of bubbles provided that the acoustic intensity is kept low (<550 W/cm^2^). These results suggest that PSNE could eventually improve the efficiency of HIFU-mediated thermal ablation of solid tumors, thus potentially making bubble-enhanced HIFU a viable option for cancer therapy.

## Competing interests

The authors declare that they have no competing interests.

## Authors’ contributions

The experiments and data analysis described in this study were carried out by PZ and JAK. PZ prepared the initial draft, which was revised by JAK and TMP. TMP conceived the study and provided his expertise and support. All authors read and approved the final manuscript.
